# The α2,3-Sialyltransferase Encoded by Myxoma Virus Is a Virulence Factor that Contributes to Immunosuppression

**DOI:** 10.1371/journal.pone.0118806

**Published:** 2015-02-23

**Authors:** Bérengère Boutard, Sophie Vankerckhove, Nicolas Markine-Goriaynoff, Mickaël Sarlet, Daniel Desmecht, Grant McFadden, Alain Vanderplasschen, Laurent Gillet

**Affiliations:** 1 Immunology-Vaccinology, Department of Infectious and Parasitic Diseases, Faculty of Veterinary Medicine, FARAH, University of Liège, Liège, Belgium; 2 Pathology, Department of Morphology and Pathology, Faculty of Veterinary Medicine, FARAH, University of Liège, Liège, Belgium; 3 Department of Molecular Genetics and Microbiology, College of Medicine, University of Florida, Gainesville, Florida, United States of America; Lisbon University, PORTUGAL

## Abstract

Myxoma virus (MYXV) induces a lethal disease called Myxomatosis in European rabbits. MYXV is one of the rare viruses that encodes an α2,3-sialyltransferase through its M138L gene. In this study, we showed that although the absence of the enzyme was not associated with any *in vitro* deficit, the M138L deficient strains are highly attenuated *in vivo*. Indeed, while all rabbits infected with the parental and the revertant strains died within 9 days post-infection from severe myxomatosis, all but one rabbit inoculated with the M138L deficient strains survived the infection. In primary lesions, this resistance to the infection was associated with an increased ability of innate immune cells, mostly neutrophils, to migrate to the site of virus replication at 4 days post-infection. This was followed by the development of a better specific immune response against MYXV. Indeed, at day 9 post-infection, we observed an important proliferation of lymphocytes and an intense congestion of blood vessels in lymph nodes after M138L knockouts infection. Accordingly, in these rabbits, we observed an intense mononuclear cell infiltration throughout the dermis in primary lesions and higher titers of neutralizing antibodies. Finally, this adaptive immune response provided protection to these surviving rabbits against a challenge with the MYXV WT strain. Altogether, these results show that expression of the M138L gene contributes directly or indirectly to immune evasion by MYXV. In the future, these results could help us to better understand the pathogenesis of myxomatosis but also the importance of glycans in regulation of immune responses.

## Introduction

The glycome of a biological entity has been defined as all the sugars it produces, including glycans linked on proteins, lipids or DNA. After nearly one-hundred years history of glycobiology, it has now become clear that glycans are perhaps one of the most important molecular components of the cells. In particular, glycans confer a diversity of structures and functions to proteins that is still underappreciated [1].

For millions of years, viruses have been co-evolving with their hosts. During this co-evolution process, viruses had to deal with the physiology of the host in order to overcome its barriers and defense mechanisms by mimicking, hijacking and sabotaging host biological processes in their favor. A growing list of studies has highlighted the importance of the glycome in the regulation of host–virus interactions and the ability of some viruses to manipulate the cellular and viral glycome [1, 2]. Viruses may modify the glycome by different mechanisms: *(i)* by regulating the expression of host glycosyltransferases or glycosidases and/or *(ii)* by encoding their own glycosyltransferases or glycosidases and/or *(iii)* by acquiring mutations that affect glycosylation sites or glycan binding specificities. Only a few viruses infecting vertebrates are known to encode glycosyltransferases [2]. Among these is Myxoma virus (MYXV) that encodes an α2,3-sialyltransferase [3].

The South American MYXV is the prototype of the *Leporipoxvirus* genus and is thus a member of the *chordopoxvirus* subfamily [4]. While MYXV causes a mild, benign infection in its well-adapted North and South American leporid hosts (*Sylvilagus bachmani* and *Sylvilagus brasiliensis*, respectively), the infection of the European rabbit (*Oryctolagus cuniculus*) results in fulminant myxomatosis, a systemic, lethal disease resulting in approximately 100% mortality [5]. The well characterized pathogenesis of myxomatosis has provided an instructive model to dissect the host/pathogen relationship and also to understand the co-evolution of hosts and poxvirus pathogens [6–9]. MYXV encodes multiple proteins which are dispensable for virus replication in cultured cells and whose function is to protect the virus from the antiviral responses of the immune system [4, 6]. One such candidate immunomodulator is the α2,3-sialyltransferase encoded by the M138L open reading frame.

Sialyltransferases are type II membrane proteins that catalyze the transfer of sialic acid from the nucleotide donor CMP- N-acetylneuraminic acid to acceptor oligosaccharides found on glycoproteins, glycolipids and polysaccharides [10, 11]. In general, sialic acids are found at the terminal positions of sialylated glycoconjugates where they have roles in: *(i)* immunological recognition or masking of antigens, *(ii)* initiation of inflammatory response, *(iii)* cell specific adhesion events, *(iv)* virus attachment or *(v)* protein stability [12].

Enzymatic properties of the α2,3-sialyltransferase expressed by MYXV have been studied in details [3, 11, 13]. It has been shown that this enzyme possesses a very broad acceptor specificity that is not found among the mammalian or bacterial α2,3-sialyltransferases. Acceptors include not only type I (Galβ1-3GlcNAcβ), type II (Galβ1-4GlcNAcβ) or type III (Galβ1-3GalNAcβ) disaccharides but also fucosylated Lewis^a^ and Lewis^x^ [11]. However, very few data are available about the roles of this protein during the viral cycle *in vitro* or *in vivo*. Firstly, it has been shown that disruption of the M138L gene caused attenuation *in vivo* [3]. Secondly, another study showed that the M138L gene product contributes to post-translational modification of the viral anti-inflammatory protein SERP-1, though this had no apparent effect upon the kinetics of *in vitro* proteinase inhibition by SERP-1 [14]. In this study, we compared *in vitro* and *in vivo* MYXV strains expressing or not the M138L gene.

## Materials and Methods

### Cells

RK13 cells (Rabbit Kidney epithelial cells, ATCC CCL-37) were cultured in DMEM (Dulbelcco’s Modified Eagle Medium, Sigma) supplemented with 10% (v/v) Fetal Calf Serum (FCS, Sigma), 2% (v/v) penicillin (100 IU/mL)—streptomycin (100 μg/mL) (Sigma) and 1% (v/v) non-essential amino acids.

### Viral strains

**Fig 1:**
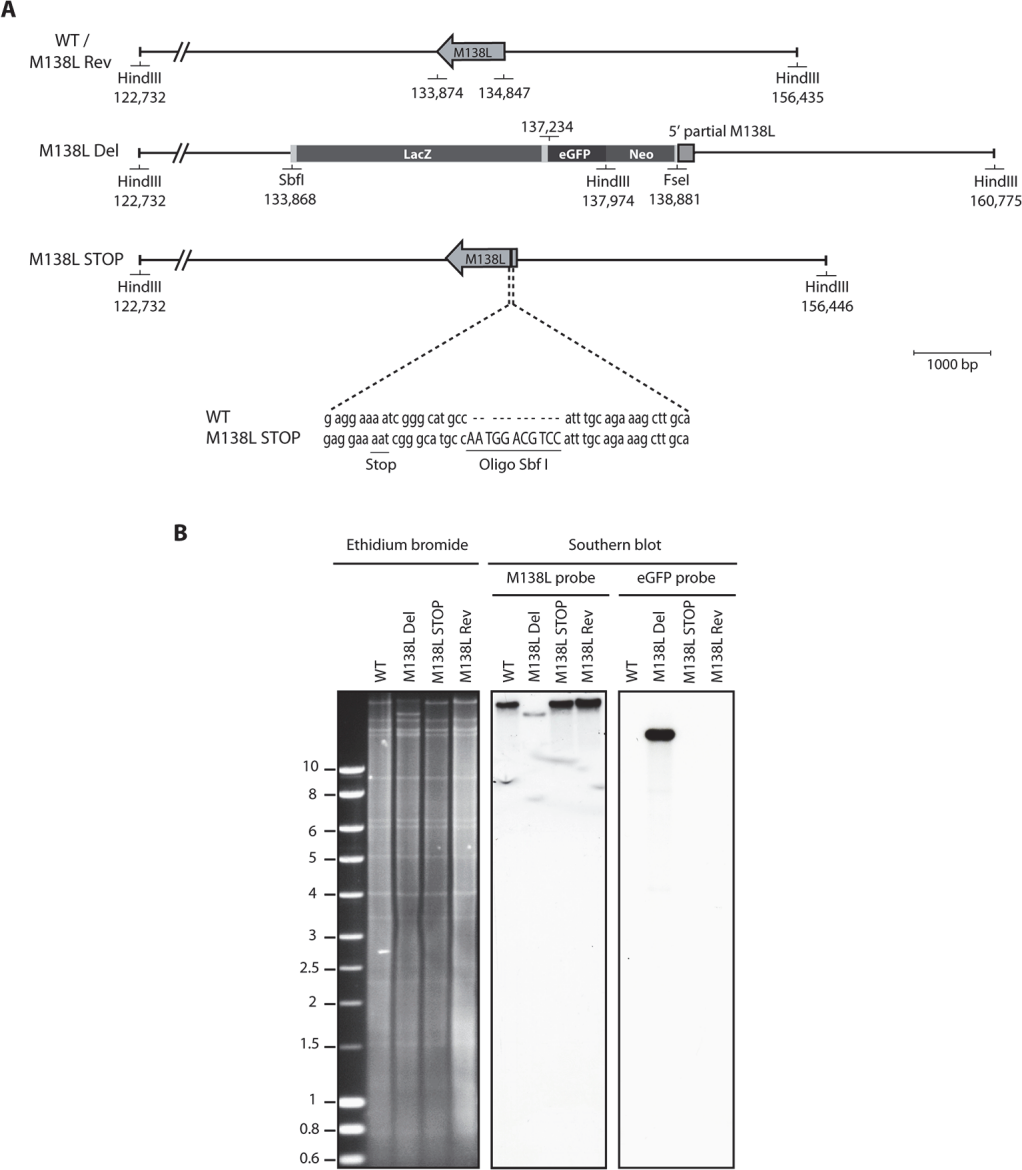
Construction of the Myxoma virus M138L mutant strains. The M138L Deleted (M138L Del), M138L Revertant (M138L Rev) and M138L STOP strains of MYXV were constructed from the hypervirulent strain Lausanne (WT). **A.** In the M138L Del strain, most of the M138L coding sequence was replaced by an eGFP-Neo cassette where eGFP is expressed as a fusion protein with the neomycin resistance protein under a synthetic early/late promoter of vaccinia virus (this cassette also contains the LacZ gene). The revertant strain was constructed by replacing the eGFP-Neo cassette in the M138L Del strain by WT sequence. The M138L STOP strain was constructed by introducing a SbfI restriction site in order to create a shift in the reading frame of the M138L gene leading to several STOP codons and to identify this mutant strain. **B.** Verification of the molecular structure of the mutant viruses by electrophoresis and Southern blotting. Viral DNA was digested with HindIII, resolved by agarose gel electrophoresis, transferred on a nitrocellulose membrane, hybridized with 32P-labeled probes, corresponding to either a fragment of M138L or eGFP and finally submitted to autoradiography. For the M138L probe, the 33,703-bp WT band becomes 22,801-bp for the M138L Del mutant and 33,714-bp for the M138L STOP strain. For the eGFP probe, the band for the M138L Del mutant is 15,242-bp. Marker sizes in Kbp are indicated on the left.

Two M138L deficient strains and a revertant strain have been constructed from the hypervirulent wild type (WT) Lausanne strain (GenBank: AF170726). In the first construct, M138L Del, most of the M138L gene was replaced by an eGFP expression cassette (this cassette also contains the LacZ gene and a neomycin resistance gene). eGFP expression is driven by a poxvirus synthetic early/late promoter [15]. In a second construct, M138L STOP, a SbfI restriction site was inserted into the M138L ORF generating a premature stop codon. Finally, a M138L revertant strain was made from the M138L Del strain (Fig. 1A). For all the constructs, upstream (132,877–133,877) and downstream (134,746–135,746) hybridizing sequences were amplified by PCR using the WT strain as template and the following primers M138LAMSENS 5’-CCATGCATCCTAGGCGACATGGTGGACGATTTTGG-3’ and M138LAMREV 5’-GGCCTGCAGGTTTATTCACTATTTCGCAAGCCTACCG-3’ for the upstream arm, and M138LPMSENS 5’- GGGCTAGCTTAAGGCCGGCCTTCTAACAGACGACGTATCTGC-3’ and M138LPMREV 5’- GGACCGGTCTTAAGCTTCAACCAGGTGACTAAGACG-3’ for the downstream arm. For the M138L Del construct, these amplification products were cloned into the pVKOV-eGFP plasmid on both sides of the eGFP expression cassette generating the pVKOV-M138L Del plasmid. Then, RK13 cells infected by the WT virus were transfected with this plasmid with FuGENE (Promega) and multiple rounds of foci purifications based on eGFP expression were performed until pure M138L Del virus was isolated. For the M138L Rev strain, the WT M138L sequence was inserted between the AM and PM arms in the pVKOV-M138L Del plasmid generating the pVKOV-M138L Rev. For the M138L STOP strain, a SbfI site was introduced in the pVKOV-M138L Rev plasmid after bp corresponding to the 134,601 bp of the Lausanne strain generating the pVKOV-M138L STOP. Then, cells infected by the M138L Del virus were transfected with either pVKOV-M138L Rev or pVKOV-M138L STOP and the M138L Rev and M138L STOP strains were purified under agar based on non-fluorescent foci formation. We also used a WT MYXV that expresses eGFP under the control of a synthetic vaccinia virus early-late promoter [16].

### Preparation of viruses

Viral strains were produced on RK13 cells infected at a MOI of 0.1 PFU per cell. When a cytopathogenic effect of +/- 80% was observed, cells and supernatants were harvested and ultracentrifuged (54,000 *g*, 2h, 4°C). The pellet was resuspended in 1.5 mL of TE9 (10 mM Tris (2-amino-2-hydroxymethyl-1,3-propanediol) pH 9, 1 mM EDTA (EthyleneDiamineTetraacetic Acid)), submitted to 3 rounds of freezing (-80°C) and thawing (37°C) and then sonicated for 1 min. Cell debris were removed by centrifugation (300 *g*, 10 min). Mature virions (MVs) were then sedimented through a 30% sucrose cushion (w/v) at 100,000 *g* for 1h, the supernatant was removed and the pellet was resuspended in PBS and stored at -80°C. Fresh EVs (extracellular virions) were prepared from RK13 cells infected at 0.8 PFU/cell. The culture supernatant was harvested 18h p.i., centrifuged to remove detached cells and cell debris (300 *g*, 20 min, 4°C). A fresh EVs preparation was produced before each experiment and used immediately without any freezing. Each EVs preparation was titrated in parallel with the experiment. For both MVs and EVs preparations, the quantity of infectious viral particles was determined by titration on RK13 cells. 6-well plates with 4.10^5^ RK13 cells per well were infected with serial dilutions of samples. After 1h of adsorption, the cells were overlaid and incubated in medium containing 0.6% CMC (carboxymethylcellulose medium viscosity, Sigma). After 6 days, cells were fixed with 5% (v/v) formaldehyde, stained with crystal violet (0.3% (w/v) crystal violet, 5% (v/v) ethanol) and foci were counted.

### Viral DNA extraction

10^6^ RK13 cells were infected at a MOI of 1 PFU/cell for 24h. Medium was removed and 1 mL of lysis buffer pH 8 was added (1.2% (w/v) SDS (Sodium Dodecyl Sulphate), Tris-HCl 50 mM pH 8, EDTA 4 mM pH 8, CaCl_2_ 4 mM, 0.4 mg/mL proteinase K). After 4h at 37°C, the cell lysate was extracted twice with phenol-chloroform and nucleic acids were precipitated with ethanol. The DNA pellet was then resuspended in deionized water and stored at -20°C.

### Restriction profile and southern blot

Viral DNAs from the 4 strains were digested with the restriction enzyme HindIII. The restriction fragments obtained were separated by electrophoresis, and the restriction profiles were analyzed after ethidium bromide staining. Fragments were transferred on a nitrocellulose membrane (Amersham Hybond-XL blotting (GE Healthcare)) under alkaline condition (NaOH 0.4M, NaCl 0.6M) as previously described [17]. The M138L probe used for hybridization was an 853 bp fragment amplified with the primers M138L-start-wt (5’-CGTCTTATTTTGGATCATACG-3’) and M138L-stop-wt (5’-GTATTTCTTTAAACGATGCACG-3'). For the eGFP probe, the relevant NheI-XhoI fragment was cut from pEGFP-C1 and gel purified. Probes were labelled with α-[^32^P]dCTP (Perkin Elmer) by using the random-primed DNA labelling kit (Roche). The membranes were hybridized with the probes at 65°C for 18h (hybridization solution: 0.25 M sodium phosphate buffer pH 7.2, 7% (w/v) SDS, 5 mg DNA of salmon sperm (Invitrogen) denatured by heating at 100°C for 5 min), washed 30 min with the first washing solution (20 mM sodium phosphate buffer pH 7.2, 5% (w/v) SDS) and 30 min with the second washing solution (20 mM sodium phosphate buffer pH 7.2, 1% (w/v) SDS), and used to expose an Amersham Hyperfilm MP (GE Healthcare).

### Isolation of peripheral blood mononuclear cells (PBMCs)

Rabbit blood samples were taken and PBMCs were separated by a Ficoll density gradient (Ficoll-Paque Plus, GE Healthcare) as described previously [18]. When necessary, PBMCs were cultured in RPMI medium (Roswell Park Memorial Institute, Sigma) containing 10% (v/v) FCS, 2% (v/v) penicillin-streptomycin, 1% (v/v) non-essential amino acids and 1% (v/v) β-mercaptoethanol (Sigma).

### Growth curves

For each virus, 6-well plates with 5.10^5^ RK13 cells per well were infected at a MOI of 0.01 PFU per cell. Every 24h, cells and supernatants were harvested together and aliquoted, to obtain 7 samples per virus (from day 0 to day 6). Growth curves were also performed on PBMCs (5.10^6^ cells per well) isolated as described above, infected *ex vivo* with the four viral strains at a MOI of 0.05 PFU per cell. The quantity of infectious viral particles was determined by titration on RK13 cells, as described above.

### Neutralization of the virions by the *Maackia amurensis* lectin

Purified MVs were diluted in DMEM and mixed (1:2, v/v) with *Maackia amurensis* (MAA) lectin (EY laboratories) diluted in DMEM. After incubation for 2 h at 37°C, virions were then bound to RK13 cells for 2h at 37°C and medium containing CMC (0.6% final concentration) was finally added. After 6 days, cells were fixed with formaldehyde, stained with crystal violet and foci were counted.

### Neutralization of the virions by serum

The sensitivity of purified MVs or fresh EVs to either neutralization by naïve serum (testing the sensitivity of virions to the alternative pathway of complement) or by immune serum was investigated. For that purpose, serum was taken from naïve or MYXV infected rabbits respectively and stored at -20°C. Sonicated purified MVs or fresh EVs were diluted in ice-cold DMEM and mixed with serum diluted in DMEM. For testing the alternative pathway of complement, heat-inactivated serum (56°C, 30 min) was used as control. After incubation for 2 h at 37°C, virions were then bound to RK13 cells for 2 h at 37°C. Unbound virions were washed away with PBS, and cells were incubated for 6 days in medium containing 0.6% CMC. After 6 days, cells were fixed with formaldehyde, stained with crystal violet and foci were counted.

### Animals

Specific pathogen free male New-Zealand White rabbits (CER, Marloie) were used, according to the directive of the European Convention for the protection of experimental vertebrate animals (CETS n°123). The protocol was approved by the Ethic Commission on the experimental animals of the University of Liège (protocol n°447). An intradermal injection of 100 PFUs of each of the 4 purified viral strains in PBS was performed in the left flank on at least 4 rabbits per strain. 4 rabbits were injected with PBS (Mock) as control. Blood sampling was performed after sedation with fentanyl (0.05 mg/kg) and acepromazine (1 mg/kg) administered intramuscularly. Rabbits were monitored twice daily to check clinical signs of myxomatosis. Clinical scores criteria were established to determine a humane end-point at which rabbits must be euthanized. Those criteria were based on the posture, water and food intakes, attitude, hydration status, eyes and nose status, general examination (breath, heart rate, temperature…) and the presence of secondary myxoma. When rabbits showed significant respiratory distress, or no food or water intake for 48 h, or a clinical score of 15 for two consecutive days, they were euthanized. Rabbits were euthanized by a lethal intravenous injection of sodium pentobarbital (100 mg/kg) after anesthesia with a combination of ketamine (35 mg/kg), xylazine (5 mg/kg) and acepromazine (1 mg/kg) administered intramuscularly.

### Histology

Organs were fixed in 4% (v/v) formaldehyde buffered in PBS, embedded in paraffin wax and cut with a microtome in regular sections of 5 μm. A haematoxylin-eosin staining was made and histological sections were examined. Importantly, rabbit neutrophils are more properly termed heterophils because they do not stain as neutral using Romanowski stains. The cytoplasm contains both small acidophilic granules and large eosinophilic granules, however they function similarly to neutrophils from other species. We have therefore called these cells neutrophils.

### Viral genome detection by Real time-PCR

DNA was purified with the QIAmp DNA Mini kit (Qiagen) from PBMCs of infected rabbits that have been collected by blood sampling at days 0, 4 and 9 post-infection. A fragment of 109 bp corresponding to a part of the M034L gene was amplified with the primers 5’-CCCGCCGACTCCTTTGTG-3’ and 5’-CGAGTTGTTAACGGACGAACG-3’. Comparable amounts of DNA were used based on concentrations measured with Nanodrop 1000 (Thermo scientific). Amplifications of quantitative PCR were made in the iCycler system (Biorad) with the supermix IQ SYBRgreen (Biorad) under the following conditions: an initial step at 95°C for 10 min, followed by 50 cycles at 95°C for 1 min, 50 cycles at 60°C for 30 seconds (sec) and 50 cycles at 72°C for 30 sec. Cellular DNA was quantified in parallel by amplifying part of the beta-globin gene (forward primer 5′-ggtatcctttttacagcacaac-3′, reverse primer 5′-CAGGTCCCCAAAGGACTCG-3′). The latter PCR products were quantified by hybridization with a TaqMan probe (5'-6-FAM-cctgggctgttttcattttctcagg-BHQ1-3’) and converted to genome copies. PCR amplifications were performed under the following conditions: initial activation of the *Taq* polymerase (Bio-Rad) at 95°C for 3 min followed by 45 cycles at 95°C for 30 sec, 55°C for 45 sec and 72°C for 45 sec. For these two reactions, the standard curves were prepared from purified viral DNA and cloned beta-globin template respectively. The total DNA concentration of standard curve samples was adapted to those of unknown samples by addition of rabbit genomic DNA. Standard curves were run with each plate.

### Flow cytometry

1.10^6^ rabbit PBMCs / well were infected *ex vivo* with the WT eGFP or M138L Del strains (both expressing eGFP). 24h after infection, cells were stained for 40 min on ice with anti-CD3 (mouse IgG1, MCA1477, AbD Serotec), anti-CD4 (mouse IgG2a, MCA799, AbD Serotec), anti-CD8 (mouse IgG1, MCA1576GA, AbD Serotec), anti-CD14 coupled with Pacific Blue (mouse IgG2a, MCA1568PB, AbD Serotec) or anti-IgM (mouse IgG1, MCA812GA, AbD Serotec) antibodies diluted in FACS buffer (PBS, 1% (w/v) BSA (Bovine Serum Albumin), 0.1% (w/v) sodium azide) to 1 μg/mL. After washings with FACS buffer, anti-CD3, anti-CD4 and anti-CD8 antibodies were detected with goat anti-mouse antibody coupled with Pacific Blue (Invitrogen), and anti-IgM with an anti-mouse IgG1 antibody coupled with phycoerythrin (PE, Invitrogen), for 40 min on ice. For the viability/apoptosis detection assay, the cells were also stained with annexin V-APC (BD Biosciences) and the impermeant dye 7-aminoactinomycin D (7-AAD) (Sigma) according to the recommendations of the manufacturers. Cells were analyzed with a FACS Aria cytometer (Becton Dickinson).

### Quantification of anti-MYXV antibodies by ELISA

Nunc Maxisorp ELISA plates (Nalgene Nunc) were coated for 18 h at 37°C with 0.1% Tween 20-disrupted WT virions (2.10^6^ PFUs/well), blocked in PBS/ 0.1% Tween-20/ 3% BSA, and incubated with rabbit sera (diluted 1/300 in PBS, 0.1% Tween-20, 3% BSA). Bound antibodies were detected with Alkaline Phosphatase conjugated goat anti-rabbit Ig polyclonal antibody (Sigma). Washings were performed with PBS containing, 0.1% Tween-20 and 3% BSA. p-Nitrophenylphosphate (Sigma) was used as substrate and absorbance was read at 405nm using an ELISA plate reader (Thermo).

## Results

### Production and *in vitro* characterization of MYXV M138L knockout strains

MYXV encodes and expresses a functional sialyltransferase that enhances its virulence [3]. However, until now, the characterization of the phenotype of MYXV M138L knockout strains has not been performed in details. In order to study the biological functions of M138L, we have disrupted M138L ORF by either replacing most of the M138L ORF with an eGFP expression cassette driven by a poxvirus synthetic early/late promoter (M138L Del) or by inserting a 11-bp oligonucleotide into the M138L ORF that generates premature stop codons (M138L STOP) (Fig. 1A). A revertant strain, called M138L Rev, was also constructed. The predicted molecular structures of the recombinant strains were confirmed by HindIII restriction mapping and Southern blotting (Fig. 1B), and further by DNA sequencing.

**Fig 2:**
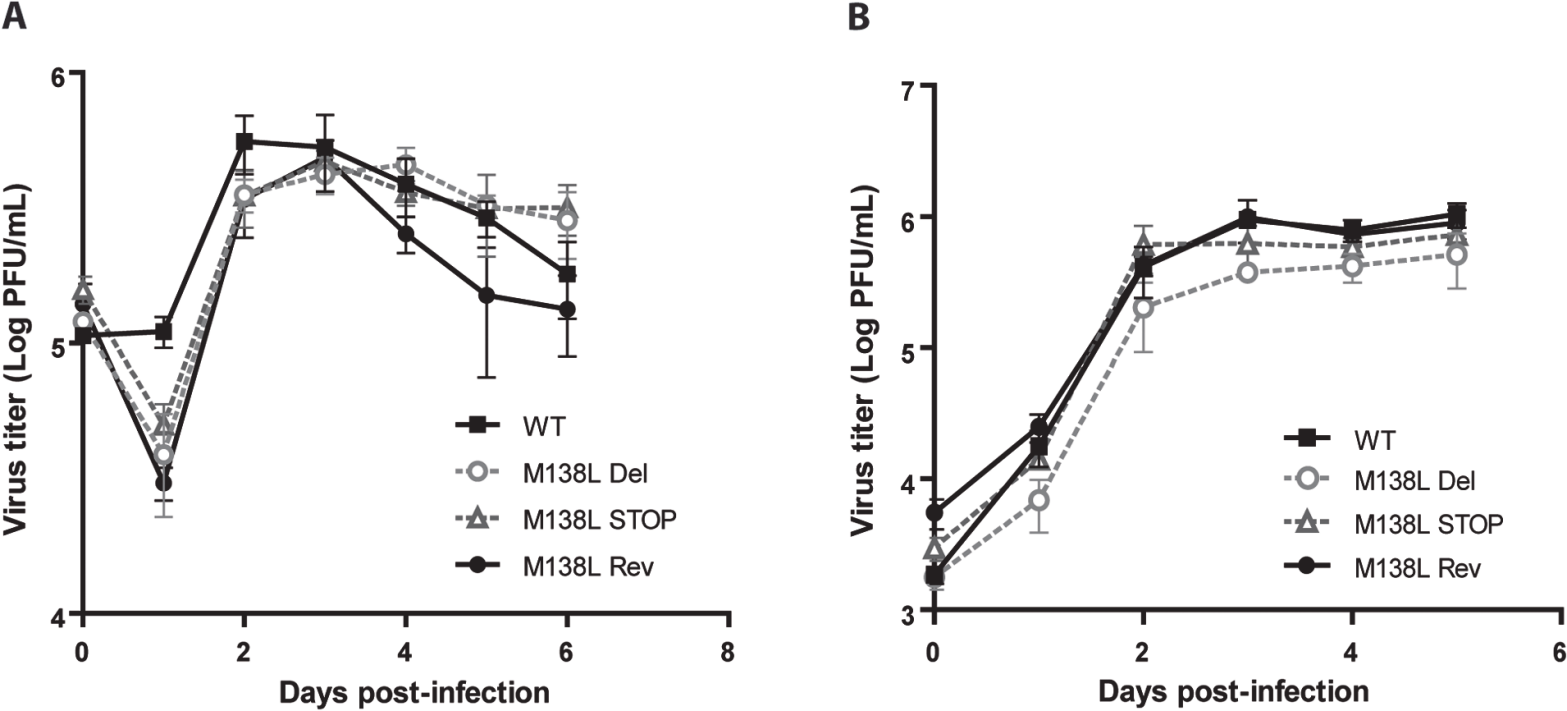
Growth of the Myxoma virus WT, M138L Del, M138L STOP and M138L Rev strains. **A.** Rabbit PBMCs were cultured *ex vivo* and infected with a MOI of 0.05. Infected cells and their supernatants were harvested together at different times post-infection and the quantity of infectious virus was determined by titration on RK13 cells. The data presented are the average +/- SEMs for 3 independent experiments. **B.** RK13 cells were infected with a MOI of 0.01 with the viral strains WT, M138L Del, M138L STOP and M138L Rev. Infected cells and their supernatants were harvested together at different times post-infection and the quantity of infectious virus was determined by titration on RK13 cells. The data presented are the average +/- SEMs for 3 independent experiments. The data were analyzed by 2way ANOVA and Bonferroni post-tests. No significant difference was observed between the different viral strains.

In order to address the roles of the M138L gene *in vitro*, WT, M138L Del, M138L STOP and M138L Rev strains were compared by multi-step growth assays on RK13 cells and on rabbit PBMCs as described [19] (Fig. 2). No defects were noted in the replication of the M138L deficient strains. Thus, the M138L gene is not essential for the virus replication *in vitro*.

**Fig 3:**
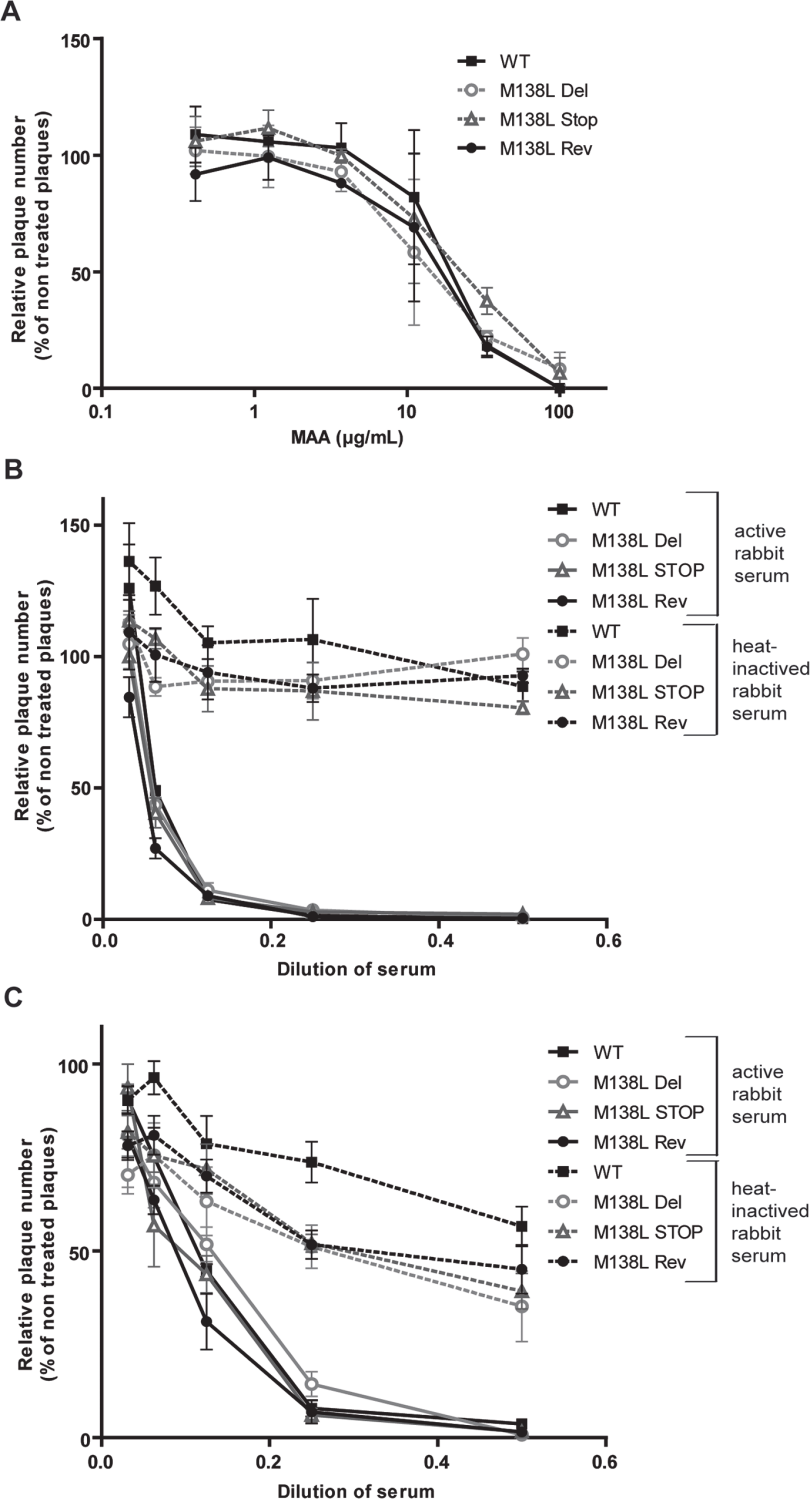
Neutralization of MYXV virions. MYXV virions were neutralized by the sialic acid-specific lectin MAA (*Maackia amurensis*) and by the alternative pathway of complement. **A.** MYXV WT, M138L Del, M138L STOP and M138L Rev mature virions (MVs) were incubated with dilutions of MAA lectin. After incubation (2h, 37°C) the viruses were plaque assayed for infectivity on RK13 cells. Data are expressed as plaque number relative to untreated equivalent virus samples. Data are the average +/- SEMs for 2 independent experiments and were analyzed by 2way ANOVA and Bonferroni post-tests. **B-C.** MYXV WT, M138L Del, M138L STOP and M138L Rev mature virions (MVs) (**B**) or freshly-prepared extracellular virions (EVs) (**C**) were incubated with serum from naïve rabbits, either untreated (continuous lines) or heat-inactivated (dashed lines). After incubation (2h, 37°C) the viruses were plaque assayed for infectivity on RK13 cells. Data are the average +/- SEMs for 3 independent experiments and were analyzed by 2way ANOVA and Bonferroni post-tests. No significant difference was observed between the different viral strains.

Sialyltransferase expression by MYXV could promote sialic acid incorporation in virions and therefore evasion of complement as observed for other viruses [20–22]. In order to test this hypothesis, we firstly compared the capacity of *Maackia amurensis* lectin (MAA), a sialic acid-specific lectin, to inhibit infection of MYXV WT, M138L Del, M138L STOP and M138L Rev strains (Fig. 3A). Surprisingly, neutralization was similar for all the strains tested. Secondly, we compared the capacity of naïve rabbit serum to neutralize these different MYXV strains through the alternative pathway of complement. Indeed, sialic acids could promote recruitment of factor H of complement. With that goal in mind, sera were collected from healthy rabbits. These sera did not contain antibodies against MYXV. These sera neutralized MVs or EVs from the different strains similarly (Fig. 3B-C). This neutralizing activity was observed only in sera in which the complement activity had been preserved. Altogether, these results indicate that M138L expression by MYXV does not modify the sensitivity of MYXV virions to neutralization by a sialic acid-specific lectin and does not confer resistance to the alternative pathway of complement.

### Effects of M138L disruption on MYXV virulence

**Fig 4:**
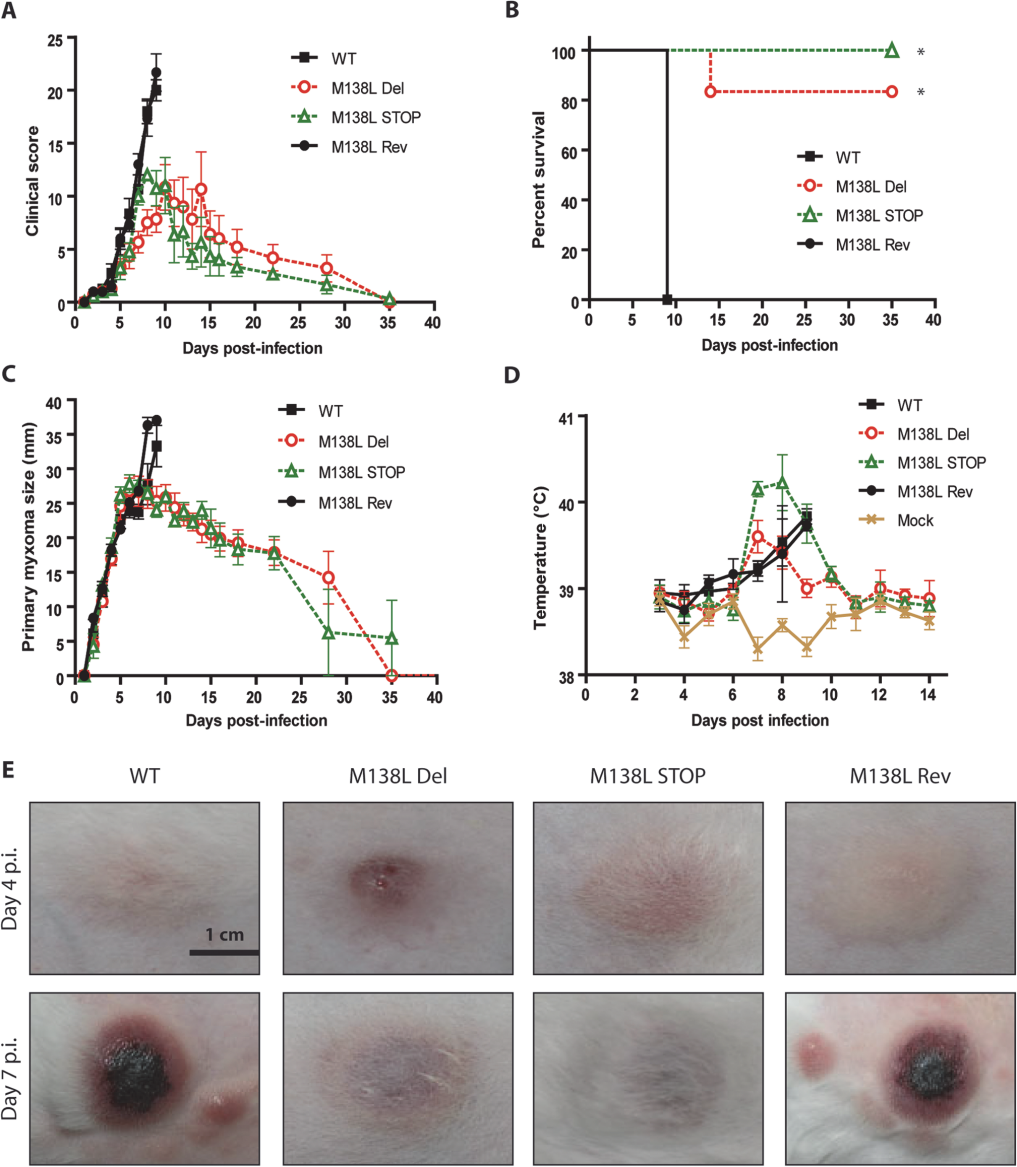
Pathogenesis of the M138L deficient strains in NZW rabbits. NZW rabbits were inoculated with 100 PFUs of Myxoma virus WT, M138L Del, M138L STOP or M138L Rev strains in the left flank. **A.** Daily clinical scores that evaluated the physical condition and clinical signs of primary and systemic infection were obtained. Clinical scores in the WT and M138L Rev groups were recorded until animals reached euthanasia criteria (significant respiratory distress, or no food or water intake for 48 h, or a clinical score of 15 for two consecutive days). **B.** The results were analyzed for survival rates among the groups. The daily percentage of survival in each group was plotted to generate the survival curve. The data were analyzed by log-rank (Mantel-Cox) test, * p<0.05. **C.** Size of the primary lesions of NZW rabbits infected with the different Myxoma virus strains. Daily measurements of the lesion area were recorded. The data presented are the average +/- SEMs. **D.** Evolution of the temperatures of the infected rabbits. The data presented are the average +/- SEMs. **E.** Pictures of primary myxomas at days 4 and 7 p.i. These pictures are representative of observations done at least on 4 rabbits. The scale bar on the first image refers to all the images.

To address the roles of the M138L gene *in vivo*, 100 PFUs of WT, M138L Del, M138L STOP or M138L Rev strains were delivered by the intradermal route of inoculation to the left flank of European rabbits. Mock infected rabbits (PBS) were used as controls. Clinical scores that reflected the rabbits' overall physical conditions and clinical signs of myxomatosis were obtained daily as described [23]. Infection with the M138L Rev strain showed a similar progression of myxomatosis and the same fatality rate (100%) as WT as these rabbits had to be euthanized at day 9 p.i. because they displayed clinical scores above 15 for two consecutive days (Fig. 4A-B, Table 1). At the same time, most of the rabbits infected with the M138L Del and STOP strains (12/13) started to recover from the attenuated signs of myxomatosis that they had presented (Fig. 4A-B, Table 1). Only one rabbit infected with the M138L Del strain had to be euthanized at day 14 p.i. due to a severe bacterial respiratory tract infection. Interestingly, analysis of clinical scores (Fig. 4A) shows that their evolutions were similar between strains until day 7 p.i. From day 8 p.i., clinical scores of rabbits infected by the M138L knockout strains started to decrease and reached zero at day 35 p.i. (Fig. 4A).

**Table 1:** Pathogenicity of the M138L knockout strains in the European rabbit.

Day	WT or M138L Rev MYXV strains	M138L Del or STOP MYXV strains
0	Inoculation of rabbits intradermally with 100 PFUs in the left flank.	Inoculation of rabbits intradermally with 100 PFUs in the left flank.
4	Primary lesions at the inoculation sites, 10–15 mm, soft and slightly necrotic in the center.	Primary lesions at the inoculation sites, 10–15 mm, soft and highly necrotic in the center.
7	Primary lesion hard, 25 mm, highly necrotic in the center. Multiple secondary lesions on body, face and ears. Bacterial infections of nasal and conjunctival mucosa.	Primary lesion hard, 25 mm but less inflammation than in WT or M138L Rev lesions. Rare secondary lesions on body, face and ears, smaller than observed with WT or Rev strains. Bacterial infections of nasal and conjunctival mucosa.
9	Severe bacterial infections in conjunctiva and respiratory tract. Secondary skin lesions turning necrotic. Rabbit sacrificed due to increasing severity of symptoms.	Bacterial infections undetected in most of the rabbits, severe in 2 of 13 rabbits. Secondary lesions rare and small.
14	-	Primary and secondary lesions regressing, bacterial infection mild to absent. 1 of 13 rabbits sacrificed due to severity of bacterial respiratory tract infection.
28	-	Absence of bacterial infections. Primary lesions consist of small, dry scabs. Small scars remain from secondary lesions.

These clinical scores reflected important differences in the size and progression of skin lesions, the onset and the severity of secondary bacterial infections and the presence of secondary lesions. Firstly, while primary myxomas grew similarly until day 7 (Fig. 4C), some primary lesions of rabbits infected with the M138L knockout strains appeared slightly more inflammatory at day 4 p.i. (Fig. 4E). In contrast, at day 7 p.i., all the rabbits infected by the M138L knockout strains displayed primary myxomas that were much less hemorrhagic and less necrotic than those from WT or M138L Rev infected rabbits (Fig. 4E). This difference was concomitant with the growth arrest of the primary lesions observed with the M138L Del or M138L STOP viruses (Fig. 4C). Interestingly, body temperatures of rabbits infected by the M138L knockout strains appeared to rise slightly faster than those of rabbits infected with WT or revertant strains although this difference was not statistically significant (Fig. 4D). This could suggest that the inflammatory response observed with the M138L knockout strains is more pronounced at the earlier time points. Finally, rabbits infected with the M138L knockout strains displayed fewer (if any) and smaller secondary lesions than rabbits infected with the WT or M138L Rev strains that displayed numerous skin secondary lesions throughout the body (nose, eyelids, ears, …). Most of the rabbits infected by the WT or M138L Rev strains showed symptoms of bacterial colonization of the respiratory tract or conjunctiva (Table 1). Altogether, these symptoms of myxomatosis were severe enough to require euthanasia of all WT and M138L Rev infected rabbits at day 9 p.i. In contrast, only one rabbit infected by a M138L knockout strain became moribund and had to be euthanized at day 14 p.i. The remaining 12 rabbits infected by the M138L Del or M138L STOP strains, completely recovered by day 35 p.i. (Fig. 4A).

### Expression of M138L by MYXV contributes to interference with the cellular immune response

**Fig 5:**
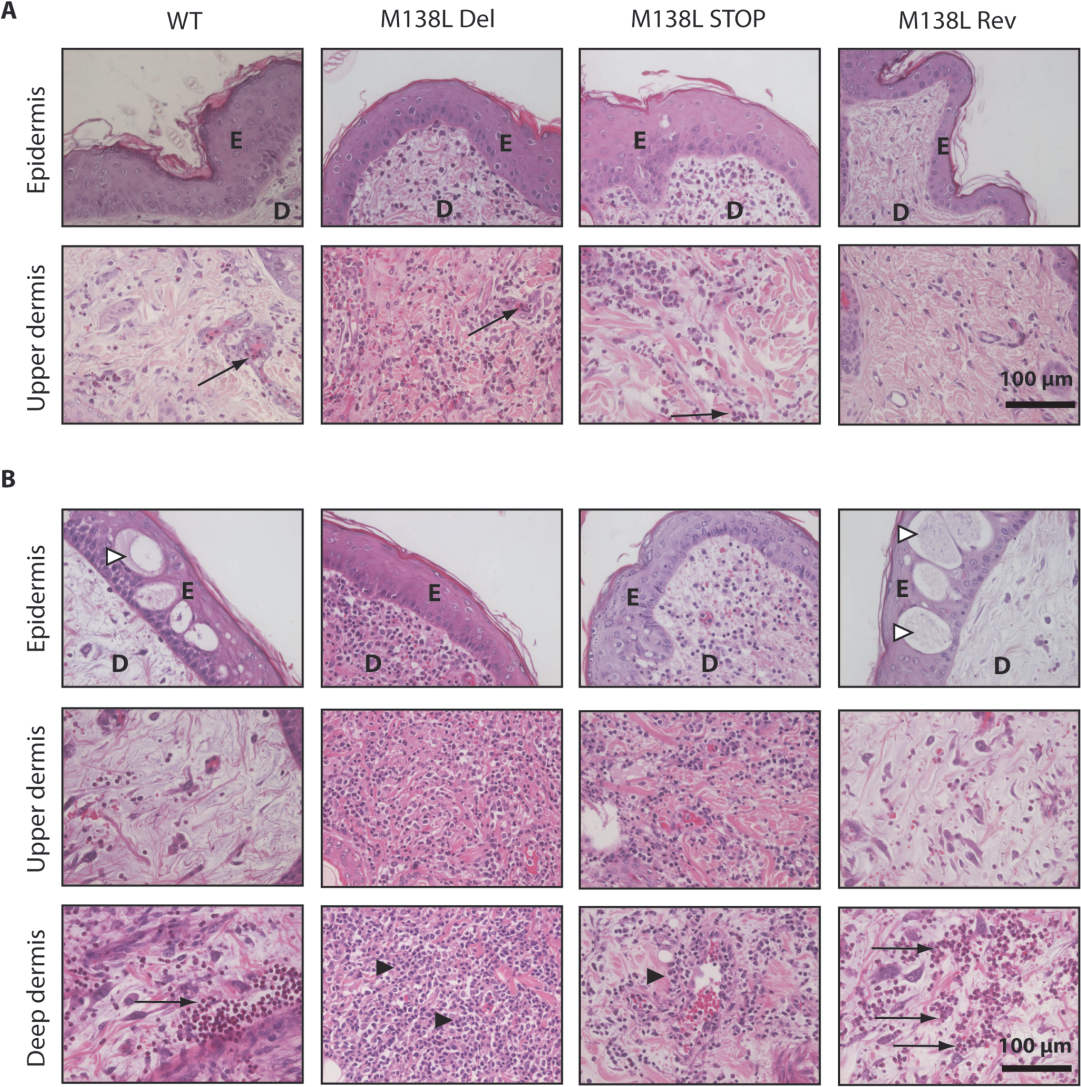
Histological differences at the primary myxoma site from rabbits infected by the Myxoma virus strains. NZW rabbits were inoculated with 100 PFUs of Myxoma virus WT, M138L Del, M138L STOP or M138L Rev strains in the left flank. **A.** Haematoxylin-eosin staining at day 4 p.i. **B.** Haematoxylin-eosin staining at day 9 p.i. These pictures are representative of observations done at least on 4 rabbits. E: epidermis, D: dermis. Black arrows show neutrophils, black arrowheads show mononuclear cells and white arrowheads show ballooning degeneration.

A detailed histological analysis was performed on tissues harvested in the time course of the experiment described above (summarized in Table 2). At day 4 p.i., epidermal hyperplasia was observed in the four groups (Fig. 5A). In the dermis, edema, widespread collagenous degeneration, myxomatous stroma infiltration and congestion were observed. Primary site of infection of rabbits infected with the M138L Del and STOP strains exhibited striking differences in the cellular infiltrates compared to rabbits infected with the WT and M138L Rev strains. Indeed, while a mild focal inflammatory response with infiltrating neutrophils was visible in the dermal tissue of the WT and M138L Rev primary lesions, very few inflammatory cells were present in the region of the dermis adjacent to the epidermal hyperplasia (Fig. 5A). In contrast, an intense neutrophil infiltration was observed throughout the dermis of the lesions generated by the M138L Del and M138L STOP strains (Fig. 5A). At day 9 p.i., although hyperplasia was still observable in all groups, ballooning degeneration was only found in WT and M138L Rev primary lesions (Fig. 5B). For these groups, a cellular inflammatory reaction was observed. However, approximately ninety percent of the cells infiltrating the dermis were neutrophils and most of them were localized in the deep dermis (Fig. 5B). Moreover, a lot of these cells appeared necrotic. By contrast, in the M138L Del and STOP primary lesions, an intense and widespread mononuclear cell infiltrate was present and neutrophils were almost absent. Strikingly, in these groups, severe inflammatory infiltrates were observed adjacent to sites of ulceration or myxomatous cells and accumulation of inflammatory cells within the deeper dermis was not observed (Fig. 5B). These results therefore suggest that M138L expression by MYXV contributes to a block of innate immune cell migration into the primary site of viral replication. Consequently, it also appears to block the subsequent recruitment of specific lymphocytes as M138L deletion was associated with an intense and widespread mononuclear cell infiltrate throughout the dermis at day 9 p.i.

**Table 2:** Major histological differences between European rabbits infected by the WT and M138L Rev strains of the Myxoma virus and the M138L knockout strains.

Day	WT or M138L Rev MYXV strains	M138L Del or STOP MYXV strains
4	*Primary sites*	*Primary sites*
	Epidermal hyperplasia, dermal edema and congestion. Mild focal inflammatory reaction with sparse number of infiltrating neutrophils visible in the dermal tissues below areas of viral replication.	Epidermal hyperplasia, dermal edema and congestion. **Intense neutrophil infiltration throughout the dermis.**
	*Lymph nodes*	*Lymph nodes*
	Lots of neutrophils. High number of apoptotic bodies.	Mainly lymphocytes. Presence of germinal centers.
9	*Primary sites*	*Primary sites*
	Epidermal hyperplasia with ballooning degeneration and micro-vesicle formation. Acute coagulation necrosis of epidermis and dermis, dermal edema and congestion. Intense inflammatory reaction in deep dermis with neutrophils dominating (90%).	Epidermal hyperplasia. Coagulation necrosis of epidermis and dermis, dermal edema and congestion. **Intense, widespread mononuclear cell infiltrate throughout the dermis. Neutrophils almost absent.**
	*Lymph nodes*	*Lymph nodes*
	Architecture disorganization. Edema and neutrophils. Nearly no lymphocytes	**Intense congestion. Lots of germinal centers.** No neutrophils.

**Fig 6:**
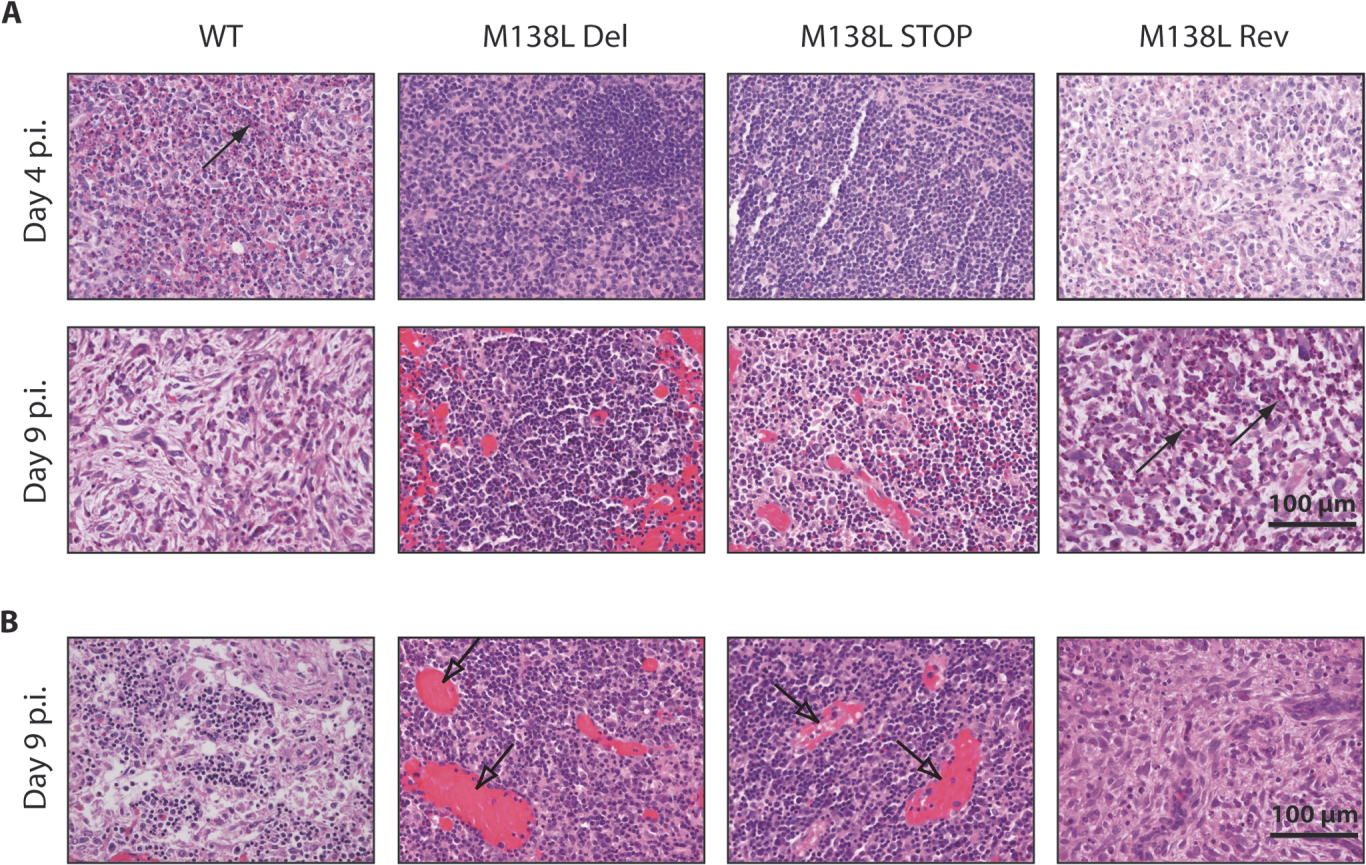
Histological differences of lymph nodes from rabbits infected by the Myxoma virus strains. **A.** Haematoxylin-eosin staining of the draining lymph nodes at days 4 and 9 p.i. **B.** Haematoxylin-eosin staining of popliteal lymph nodes at day 9 p.i. These pictures are representative of observations done at least on 4 rabbits. Black arrows show neutrophils and transparent arrows show congestion of blood vessels.

Very striking differences were also observed in lymphoid tissues. At day 4 p.i., a massive neutrophil and macrophage infiltration was observed in draining lymph nodes (axillary nodes [24]) from WT and M138L Rev infected rabbits (Fig. 6A). Moreover, significant necrosis of lymphocytes was evident and was associated with an absence of lymphocyte reactivity in the germinal centers. In contrast, draining lymph nodes from M138L Del and M138L STOP infected rabbits displayed no necrosis and normal lymph node architecture was observed (Fig. 6A). At day 9 p.i., most of the neutrophils were necrotic in lymph nodes from WT and M138L Rev infected rabbits. Moreover, the architecture was totally disorganized and lymphocytes were nearly absent. In contrast, draining lymph nodes from M138L Del and M138L STOP infected rabbits exhibited numerous germinal centers and intense congestion of the blood vessels (Fig. 6A). Very similar damage was observed at day 9 p.i. in popliteal lymph nodes (Fig. 6B). These results therefore suggest that animals infected by the WT or M138L Rev strains mounted a poor cellular adaptive immune response against MYXV while the adaptive response is particularly strong against the M138L knockout strains.

### Dissemination of the M138L knockout strains

The lesions observed in lymphoid organs of rabbits infected by the WT or M138L Rev strains suggest that replication of the virus in these organs and therefore dissemination could be lower in M138L knockout strains. This would be in accordance with the much lower number of secondary lesions observed in rabbits infected with the M138L Del and M138L STOP strains in comparison with the WT and M138L Rev strains.

**Fig 7:**
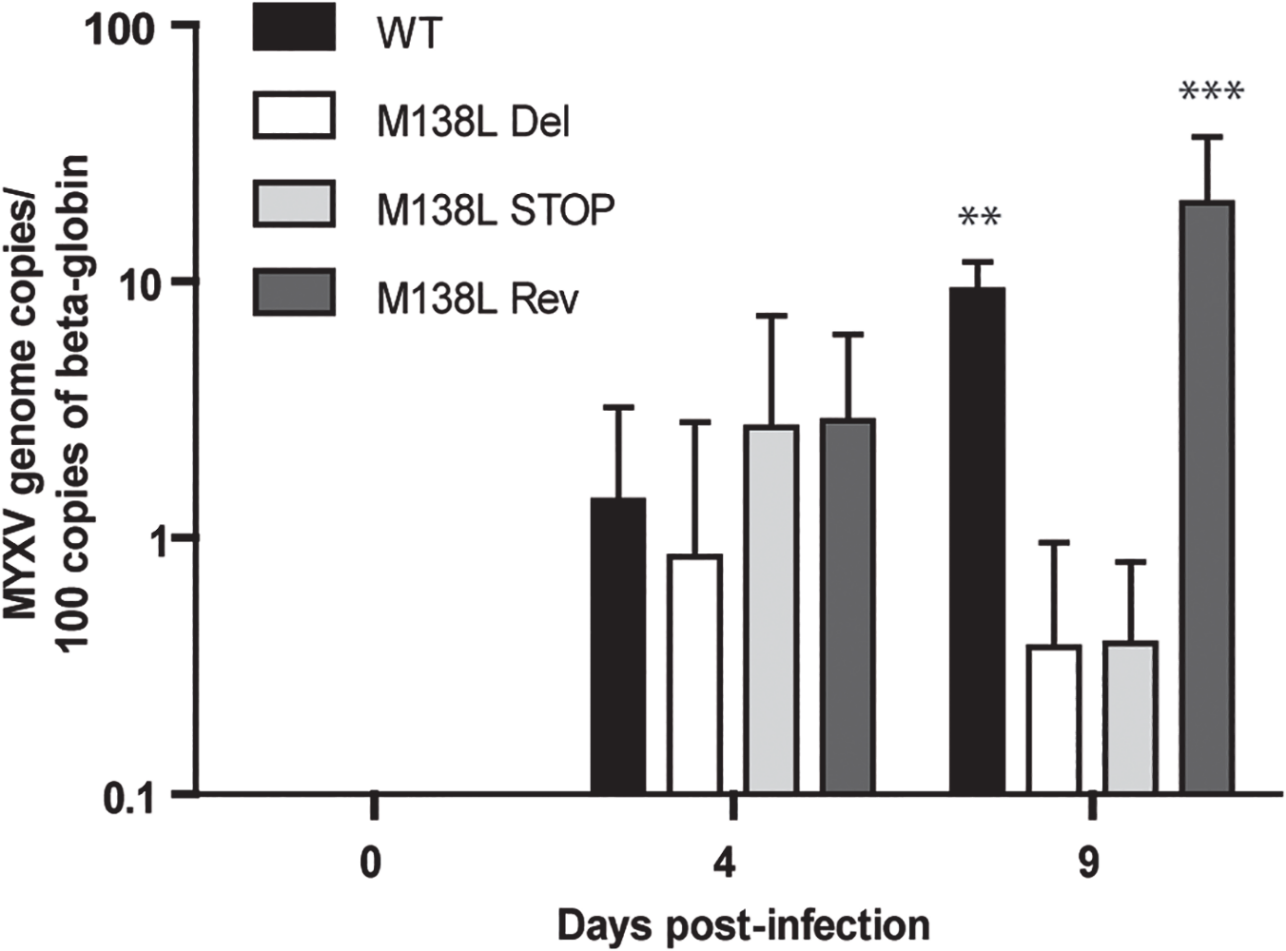
Dissemination of Myxoma virus in PBMCs during *in vivo* infection. Rabbits (minimum 4 per strain) were infected with 100 PFUs of WT, M138L Del, M138L STOP or M138L Rev strains. DNA was extracted from PBMCs (collected by blood sample) of infected rabbits at different times post-inoculation (days 0, 4 and 9). Data are expressed as the number of MYXV genome copies per 100 copies of beta-globin cellular gene. The data presented are the average +/- SEMs for 3 independent replicates and were analyzed by 2way ANOVA and Bonferroni post-tests, **p<0.01 and ***p<0.001.

**Fig 8:**
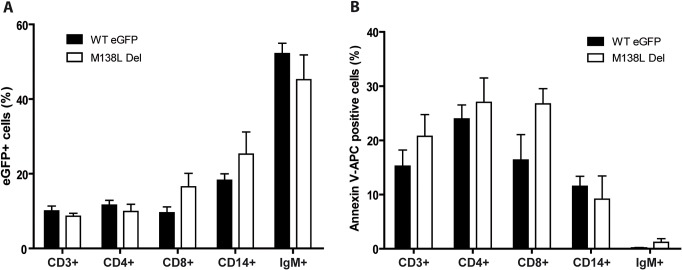
*Ex vivo* infection of rabbit PBMCs by MYXV eGFP expressing strains. PBMCs were infected with WT eGFP or M138L Del strains. 24h p.i., cells were analyzed by flow cytometry for eGFP expression (**A**) and for apoptosis by Annexin V-APC staining (**B**). The data presented are the average +/- SEMs for 3 independent replicates and were analyzed by Student's test. No statistical difference was observed between groups.

To investigate the importance of M138L expression in the dissemination of MYXV, we assayed the number of MYXV genome copies by quantitative PCR of DNA from PBMCs over time (Fig. 7). While the number of MYXV genome copies per PBMC was similar between strains at day 4 p.i., it was significantly lower in M138L knockout infected rabbits at day 9 p.i. in comparison with WT and M138L Rev infected animals. This difference could reflect a difference of tropism between the different strains. Indeed, subtle differences of sialic acid expression at the surface of the virion could allow interaction with different sialic acid binding immunoglobulins-like lectins (siglecs) at the surface of leukocytes [25]. In order to test this hypothesis, the M138L Del strain and a WTeGFP strain were used to infect PBMCs from naive rabbits *ex vivo* (Fig. 8). Twenty-four hours p.i., the number of eGFP positive cells was compared between the strains in CD3 (general T cells), CD4 (T helper), CD8 (cytotoxic T cells), CD14 (mostly monocytes) and IgM positive cells (B cells). For each cell type, no significant difference was observed between the strains (Fig. 8A). Moreover, no difference in the survival of infected cells was observed between the strains (Fig. 8B). These results therefore suggest that the main difference in the dissemination of MYXV observed between the strains could be a consequence of a better immune response raised against M138L knockout strains.

### Antibody response against the M138L knockout strains

**Fig 9:**
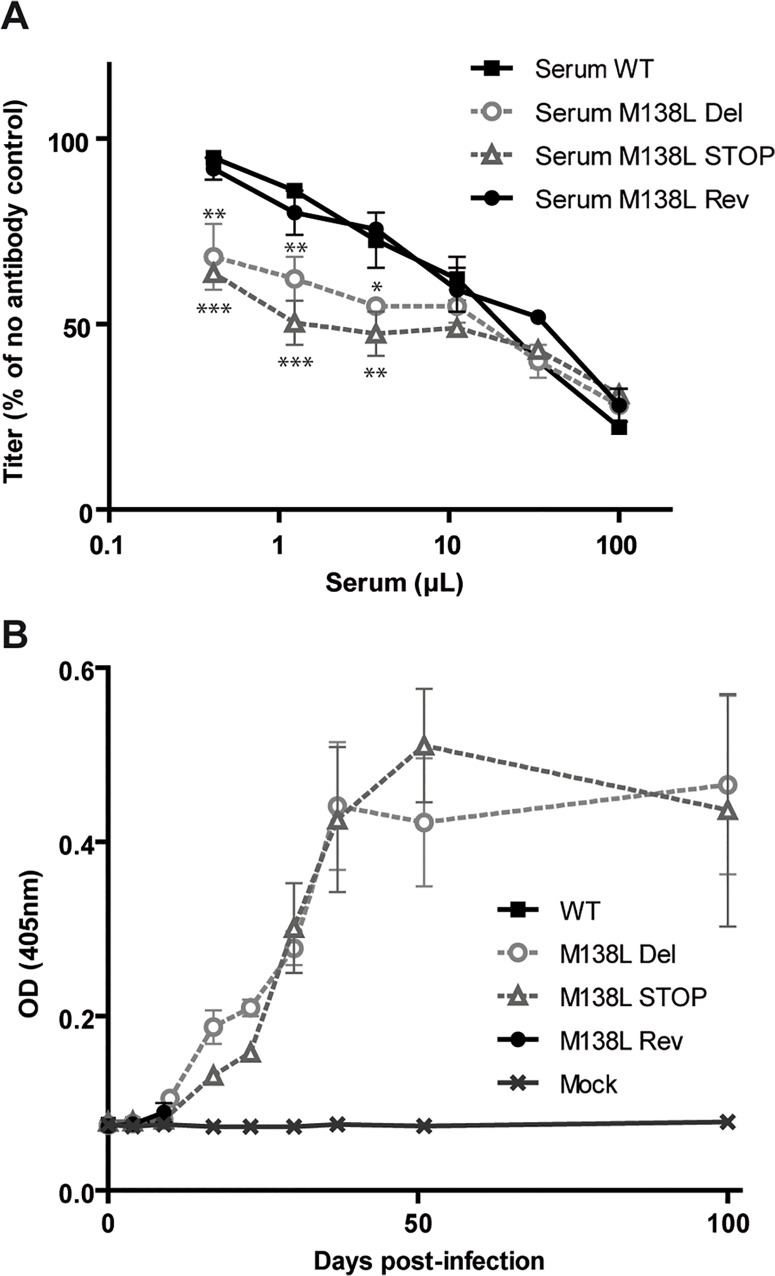
Antibody response against the M138L deficient strains in NZW rabbits. NZW rabbits were inoculated with 100 PFUs of Myxoma virus (MYXV) WT, M138L Del, M138L STOP or M138L Rev strains in the left flank or mock infected. **A.** Sera collected at day 9 p.i. were compared for their capacity to neutralize MYXV WT virions. Briefly, MYXV WT virions were incubated with various amounts of sera from the different rabbits infected by the different strains. After incubation (2h, 37°C) the viruses were plaque assayed for infectivity on RK13 cells. MYXV titers are expressed relative to virus without antibody. The data presented are the average +/- SEMs for 3 independent replicates and were analyzed by 2way ANOVA and Bonferroni post-tests, * p<0.05, ** p<0.01, *** p<0.001. **B.** The titer of anti-MYXV antibodies was estimated by ELISA as described in the Materials and Methods. Each value represents the mean +/- SEMs of the data obtained for the rabbits of each group. The sera of mock infected rabbits were taken as controls. Only samples until day 9 p.i. are shown for the rabbits infected by WT and M138L Rev strains as they had to be euthanized at day 9 post-infection.

We further compared the capacity of sera from rabbits of the different groups to neutralize MYXV WT virions (Fig. 9A). Briefly, MYXV WT virions were incubated with sera of rabbits infected with the WT, M138L Del, M138L STOP and M138L Rev taken at day 9 p.i. and then titrated on RK13 cells (Fig. 9A). Sera from M138L Del and M138L STOP rabbits neutralized significantly better MYXV WT virions than sera from the WT and M138L Rev infected rabbits. After day 9 p.i., rabbits infected by the M138L knockout strains developed a strong antibody response against MYXV (Fig. 9B).

### Challenge

Animals previously infected with either the M138L Del or M138L STOP strains were challenged with a lethal dose of WT strain (100 PFUs) 100 days post-initial infection. As expected, non-immune rabbits (Mock-WT) developed severe myxomatosis and had to be euthanized 9 days post-challenge. In contrast, M138L Del or M138L STOP infected rabbits (M138L Del—WT and M138L STOP—WT) exhibited complete resistance to the challenge (S1 Fig.). Rabbits from the two groups displayed low clinical scores and the myxomas at the site of injection stopped growing around 4 days post-challenge (S1 Fig.).

**Fig 10:**
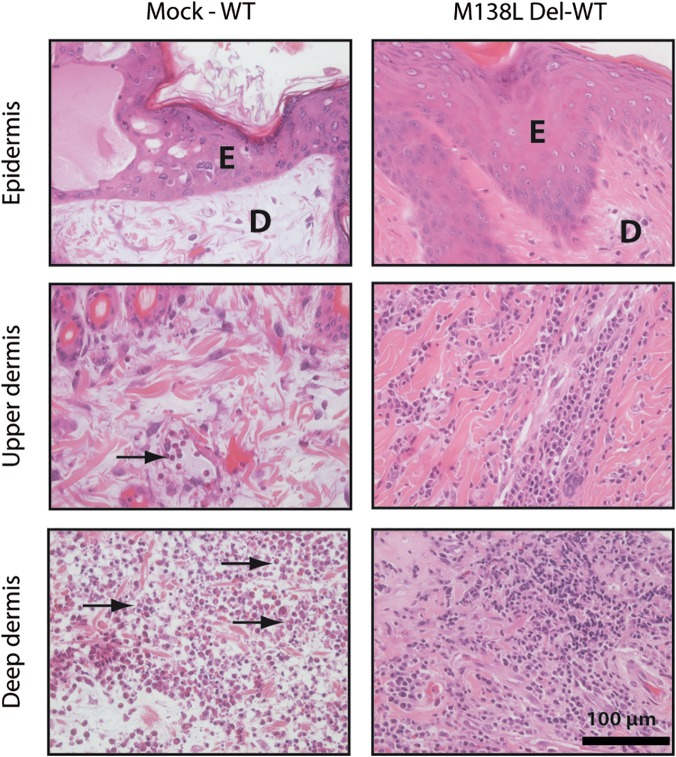
Histological differences after a challenge with 100 PFUs of the WT Myxoma virus strain. Mock infected rabbits and rabbits previously infected with the M138L Del strain 100 days before were infected with the WT strain. The primary myxoma were stained with haematoxylin-eosin staining at day 9 post-challenge. These pictures are representative of observations done at least on 2 rabbits. E: epidermis, D: dermis. Black arrows show neutrophils.

While primary myxomas that developed in the Mock-WT group displayed all the features described above for WT and M138L Rev rabbits, WT MYXV challenge of M138L knockout surviving rabbits generated very different lesions at the site of challenge. Strikingly, at day 9 post-challenge, massive infiltration of mononuclear leukocytes was observed throughout the dermis, including regions adjacent to the epidermis (Fig. 10). Moreover, draining lymph nodes from M138L Del—WT and M138L STOP—WT infected rabbits exhibited numerous germinal centers in contrast to lymph nodes from rabbits of the Mock—WT group. Altogether, these results suggest that the immune evasion mechanisms allowed by M138L expression are not effective against the recruitment of immune cells during memory response.

## Discussion

In this study, we characterized in details the phenotype of MYXV strains that do not express the M138L gene which encodes an α2,3-sialyltransferase. While we did not reveal any *in vitro* difference associated with M138L deficiency (Figs. 2 and 3), we observed that M138L knockout strains were highly attenuated *in vivo* (Fig. 4). The resistance to the infection by these strains was associated with an increased migration of neutrophils to the primary site of virus replication (Fig. 5) and the establishment of a better adaptive immune response that protected these animals against a challenge with the MYXV WT strain (Fig. 10 and S1 Fig.).

Surprisingly, our M138L deficient strains displayed a bigger *in vivo* attenuation than initially described by Jackson *et al*. [3]. Indeed, these authors showed that their M138L deficient strain (Lu(lacZ+/MST3N-)) could be classed as grade II virulence (95–99% mortality, 13 to 16 days mean survival) based on the classification of Fenner and Marshall [26]. In contrast, the two M138L deficient strains described in this study would have been classed as grade V (<30% mortality and survival time not calculable) as only 1 inoculated rabbit out of 13 died (<8%). These differences could be explained by environmental factors or differences in the sanitary status of the rabbits. Moreover, the previously described endpoint criteria [23] that we used were probably different from those used by Jackson *et al*.

Our results suggest that the M138L encoded sialyltransferase by MYXV contributes to immunosuppression by inducing, either directly or indirectly, a blockade of migration to the infected sites of some inflammatory innate immune cells, especially neutrophils. In MYXV WT and M138L Rev infections, the reduced afflux of innate sentinel cells to the site of infection likely impedes the establishment of an efficient anti-MYXV specific immune response. Interestingly, several attenuated MYXV strains induce a similar increased inflammation at the inoculation site [27, 28] showing that this is a crucial step for the development of myxomatosis.

Much evidence does exist to show that early stage innate immunity plays an important role in the establishment of adaptive immune responses. Thus, while neutrophils have long been considered as simple killer cells participating as first line of innate immune defense and as effectors of adaptive immunity, recent evidence has extended the functions of these cells [29, 30]. Indeed, several studies have showed that neutrophils participate at various levels in the shaping of the adaptive immune responses [29–32] among which those aiming at controlling the early stages of viral infections [33–35]. Therefore, during primary infection with MYXV, neutrophils could have different roles that could justify the development of mechanisms to inhibit their recruitment.

Firstly, they could exert a direct and potent antiviral effect. Thus, neutrophils restrict mouse cytomegalovirus infection in peripheral tissues through a mechanism involving TRAIL expression [34]. Another study has recently showed that neutrophils recruited to the site of infection could protect from virus infection by releasing neutrophil extracellular traps (NETs) [35]. Interestingly, one of the virus stimulus used in this study was the systemic administration of MYXV [35]. Importantly, this study showed that after MYXV infection, NETs play a role in protecting host cells from disseminating viral infection. Interestingly, we observed that the M138L knockout strains display reduced dissemination (Fig. 7).

Secondly, neutrophils could also have a role in the establishment of a specific adaptive immune response against MYXV. Indeed, neutrophils can affect adaptive immune responses by either direct or indirect interaction [36, 37]. Thus, neutrophils have been shown to affect adaptive immunity by direct effects on B and T cells [38], but also by affecting the function of dendritic cells (DCs) [30, 39–41]. Interestingly, after vaccinia virus intradermal inoculation, neutrophils were identified as the cells transporting the antigens from the dermis to the bone marrow and allowing the emergence of specific CD8+ T cells at that place [42].

How expression of an α2,3-sialyltransferase contributes to block migration of neutrophils remains unanswered. Interestingly, our results showed that this mechanism is effective during primary infection but not when an established adaptive immune memory is present as mononuclear cells, likely memory lymphocytes, invade the entire dermis at the site of challenge with the WT strain of MYXV in immune rabbits (Fig. 10). M138L appears therefore involved in blocking, either directly or indirectly, the recruitment of innate immune cells in the absence of pre-existing specific immunity.

Firstly, one chemoattractant for neutrophils is complement fragment 5a. Interestingly, surface sialic acids appear to be crucial in protecting membranes from activation of the alternative complement pathway by allowing recruitment of factor H and inhibition of cell-bound C3b [43]. Thus, some viruses such as Sindbis virus, take advantage of the expression of sialic acids by the host cell to resist complement [44] and this resistance correlates with the amounts of sialic acid that are expressed on the cells in which the virus was grown [20–22]. By increasing the presence of sialic acids at membrane surfaces, M138L expression could allow the recruitment of factor H and could therefore inhibit the activation of the alternative pathway of complement and reduce neutrophil influx. However, despite many attempts, we did not observe any effect of M138L expression on complement activation on either virions (Fig. 3) or infected cells. Moreover, what was observed was not a reduced recruitment of neutrophils from the bloodstream as these cells were able to extravasate and migrate for some distance in the dermis (Fig. 5). It therefore seems that the problem is less a defect of chemoattractant release on the site of infection than a blockade of their action.

Secondly, sialylation could affect the secretion [45], the activity or the half-life [46] of targeted proteins. MYXV possesses a vast and complex arsenal of immunomodulatory proteins [6] that could be affected by expression of its α2,3-sialyltransferase. Several of these proteins are secreted and could interfere with leukocyte chemotaxis [47]. M-T1 (M001R/L) is a secreted viral protein that has been shown to bind and inhibit chemokines of the CC-subfamily [48, 49]. However, deletion of M-T1 had no significant effects on disease progression or in the overall fatality rate of infected European rabbits [48]. M-T7 (M007R/L) is abundantly secreted and is able to bind interferon-γ as well as chemokines of the CXC-, CC- and C-subfamilies [50–53]. Interestingly, rabbits infected by a M-T7 deficient strain of MYXV displayed a dramatic reduction in disease symptoms [51] that is very similar to the one observed in this study. The potential sialylation of M-T7 and its functional consequences will therefore have to be investigated in the future. MYXV encodes also another kind of viral immunomodulatory proteins, serpins (serine protease inhibitors), which inhibit pro-inflammatory serine proteases. Interestingly, SERP-1 (M008R/L) is secreted and has been shown to interfere with inflammation in primary lesions [54]. Moreover, this is the only protein that has been shown to date to be post-translationally modified by the M138L encoded α2,3-sialyltransferase [14]. However, when we compared the progression of the disease and the lesions induced by a SERP-1 deficient strain with the M138L knockouts used in the study, we observed that, in comparison with M138L deficiency, absence of SERP-1 had only moderate effects on disease progression or in the overall fatality rate as only 30% of the infected rabbits survived (data not shown). Moreover, an important epidermal damage was observed and the immune cell infiltration was reduced in comparison with M138L deficient strains (data not shown). Therefore, even if M138L expression could have an effect through sialylation of SERP-1, it does not appear to be its main target.

Thirdly, the phenotype that we observed for the M138L knockout strains could also be independent of the sialyltransferase activity of the M138L gene product. As the structure-activity relationship of sialyltransferases in general is still not known precisely [55], a panel of different mutants for specific sites will have to be generated [56] and the association between their sialyltransferase activity and their *in vivo* phenotype could then be analyzed.

In conclusion, we showed in this study that the M138L gene of MYXV is a virulence factor that affects the pathogenesis of myxomatosis in European rabbits. Indeed, M138L deficiency was associated with an increased influx of neutrophils at the primary site of infection and with a subsequent better adaptive immune response against the virus. In the future, these results could help us to better understand some immune evasion mechanisms developed by MYXV in particular but also to better understand antiviral immunity in general.

## Supporting Information

S1 Fig Protection of the rabbits infected with the M138L knockout strains against a WT strain challenge. NZW rabbits were inoculated with 100 PFUs of MYXV M138L Del or M138L STOP strains in the left flank or left uninfected (Mock). 100 days p.i., rabbits of all groups were challenged with the inoculation of 100 PFUs of the MYXV WT strain. **A.** Daily clinical scores that evaluated the physical condition and clinical signs of primary and systemic infection were obtained. Clinical scores in the Mock vaccinated group were recorded until animals reached euthanasia criteria (significant respiratory distress, or no food or water intake for 48 h, or a clinical score of 15 for two consecutive days). **B.** The results were analyzed for survival rates among the groups. The daily percentage of survival in each group was plotted to generate the survival curve. The data were analyzed by log-rank (Mantel-Cox) test, * p<0.05. **C.** Size of the primary lesions of NZW rabbits from the different groups. Daily measurements of the lesion area were recorded. The data presented are the average +/- SEMs.
(PDF)

## References

[1] Vigerust DJ, Shepherd VL. Virus glycosylation: role in virulence and immune interactions. Trends in microbiology. 2007;15(5):211–8. 10.1016/j.tim.2007.03.003 . 17398101 PMC7127133

[2] Markine-Goriaynoff N, Gillet L, Van Etten JL, Korres H, Verma N, Vanderplasschen A. Glycosyltransferases encoded by viruses. J Gen Virol. 2004;85(Pt 10):2741–54. Epub 2004/09/28. 85/10/2741 [pii]. 10.1099/vir.0.80320-0 . 15448335

[3] Jackson RJ, Hall DF, Kerr PJ. Myxoma virus encodes an alpha2,3-sialyltransferase that enhances virulence. Journal of virology. 1999;73(3):2376–84. 9971821 10.1128/jvi.73.3.2376-2384.1999 PMC104483

[4] Stanford MM, Werden SJ, McFadden G. Myxoma virus in the European rabbit: interactions between the virus and its susceptible host. Veterinary research. 2007;38(2):299–318. 10.1051/vetres:2006054 . 17296158

[5] Kerr PJ. Myxomatosis in Australia and Europe: a model for emerging infectious diseases. Antiviral research. 2012;93(3):387–415. 10.1016/j.antiviral.2012.01.009 . 22333483

[6] Spiesschaert B, McFadden G, Hermans K, Nauwynck H, Van de Walle GR. The current status and future directions of myxoma virus, a master in immune evasion. Veterinary research. 2011;42:76 10.1186/1297-9716-42-76 21658227 PMC3131250

[7] Kerr PJ, Ghedin E, DePasse JV, Fitch A, Cattadori IM, Hudson PJ, et al Evolutionary history and attenuation of myxoma virus on two continents. PLoS pathogens. 2012;8(10):e1002950 10.1371/journal.ppat.1002950 23055928 PMC3464225

[8] Kerr PJ, Rogers MB, Fitch A, Depasse JV, Cattadori IM, Hudson PJ, et al Comparative analysis of the complete genome sequence of the California MSW strain of myxoma virus reveals potential host adaptations. Journal of virology. 2013;87(22):12080–9. 10.1128/JVI.01923-13 23986601 PMC3807925

[9] Kerr PJ, Rogers MB, Fitch A, Depasse JV, Cattadori IM, Twaddle AC, et al Genome scale evolution of myxoma virus reveals host-pathogen adaptation and rapid geographic spread. Journal of virology. 2013;87(23):12900–15. 10.1128/JVI.02060-13 24067966 PMC3838154

[10] Li Y, Chen X. Sialic acid metabolism and sialyltransferases: natural functions and applications. Applied microbiology and biotechnology. 2012;94(4):887–905. 10.1007/s00253-012-4040-1 22526796 PMC3534974

[11] Sujino K, Jackson RJ, Chan NW, Tsuji S, Palcic MM. A novel viral alpha2,3-sialyltransferase (v-ST3Gal I): transfer of sialic acid to fucosylated acceptors. Glycobiology. 2000;10(3):313–20. . 10704530 10.1093/glycob/10.3.313

[12] Varki A, Gagneux P. Multifarious roles of sialic acids in immunity. Annals of the New York Academy of Sciences. 2012;1253:16–36. 10.1111/j.1749-6632.2012.06517.x 22524423 PMC3357316

[13] Sugiarto G, Lau K, Yu H, Vuong S, Thon V, Li Y, et al Cloning and characterization of a viral alpha2-3-sialyltransferase (vST3Gal-I) for the synthesis of sialyl Lewisx. Glycobiology. 2011;21(3):387–96. 10.1093/glycob/cwq172 20978012 PMC3033747

[14] Nash P, Barry M, Seet BT, Veugelers K, Hota S, Heger J, et al Post-translational modification of the myxoma-virus anti-inflammatory serpin SERP-1 by a virally encoded sialyltransferase. The Biochemical journal. 2000;347(Pt 2):375–82. 10749666 10.1042/0264-6021:3470375 PMC1220969

[15] Chakrabarti S, Sisler JR, Moss B. Compact, synthetic, vaccinia virus early/late promoter for protein expression. BioTechniques. 1997;23(6):1094–7. . 9421642 10.2144/97236st07

[16] Johnston JB, Barrett JW, Chang W, Chung CS, Zeng W, Masters J, et al Role of the serine-threonine kinase PAK-1 in myxoma virus replication. Journal of virology. 2003;77(10):5877–88. 12719581 10.1128/JVI.77.10.5877-5888.2003 PMC154029

[17] Machiels B, Stevenson PG, Vanderplasschen A, Gillet L. A gammaherpesvirus uses alternative splicing to regulate its tropism and its sensitivity to neutralization. PLoS Pathog. 2013;9(10):e1003753 10.1371/journal.ppat.1003753 24204281 PMC3814654

[18] Machiels B, Lete C, Guillaume A, Mast J, Stevenson PG, Vanderplasschen A, et al Antibody evasion by a gammaherpesvirus O-glycan shield. PLoS Pathog. 2011;7(11):e1002387 10.1371/journal.ppat.1002387 22114560 PMC3219721

[19] Blanie S, Mortier J, Delverdier M, Bertagnoli S, Camus-Bouclainville C. M148R and M149R are two virulence factors for myxoma virus pathogenesis in the European rabbit. Veterinary research. 2009;40(1):11 10.1051/vetres:2008049 19019281 PMC2695013

[20] Hirsch RL, Griffin DE, Winkelstein JA. Host modification of Sindbis virus sialic acid content influences alternative complement pathway activation and virus clearance. Journal of immunology. 1981;127(5):1740–3. . 6117595

[21] Hirsch RL, Griffin DE, Winkelstein JA. Natural immunity to Sindbis virus is influenced by host tissue sialic acid content. Proceedings of the National Academy of Sciences of the United States of America. 1983;80(2):548–50. 6300853 10.1073/pnas.80.2.548 PMC393416

[22] McSharry JJ, Pickering RJ, Caliguiri LA. Activation of the alternative complement pathway by enveloped viruses containing limited amounts of sialic acid. Virology. 1981;114(2):507–15. . 6270885 10.1016/0042-6822(81)90230-0

[23] Liu J, Wennier S, Reinhard M, Roy E, MacNeill A, McFadden G. Myxoma virus expressing interleukin-15 fails to cause lethal myxomatosis in European rabbits. Journal of virology. 2009;83(11):5933–8. 10.1128/JVI.00204-09 19279088 PMC2681933

[24] Soto-Miranda MA, Suami H, Chang DW. Mapping superficial lymphatic territories in the rabbit. Anatomical record. 2013;296(6):965–70. 10.1002/ar.22699 . 23613262

[25] Zou Z, Chastain A, Moir S, Ford J, Trandem K, Martinelli E, et al Siglecs facilitate HIV-1 infection of macrophages through adhesion with viral sialic acids. PloS one. 2011;6(9):e24559 10.1371/journal.pone.0024559 21931755 PMC3169630

[26] Fenner F, Marshall ID. A comparison of the virulence for European rabbits (Oryctolagus cuniculus) of strains of myxoma virus recovered in the field in Australia, Europe and America. The Journal of hygiene. 1957;55(2):149–91. 13439170 10.1017/s0022172400037098 PMC2217926

[27] Best SM, Collins SV, Kerr PJ. Coevolution of host and virus: cellular localization of virus in myxoma virus infection of resistant and susceptible European rabbits. Virology. 2000;277(1):76–91. 10.1006/viro.2000.0505 . 11062038

[28] Best SM, Kerr PJ. Coevolution of host and virus: the pathogenesis of virulent and attenuated strains of myxoma virus in resistant and susceptible European rabbits. Virology. 2000;267(1):36–48. 10.1006/viro.1999.0104 . 10648181

[29] Mantovani A, Cassatella MA, Costantini C, Jaillon S. Neutrophils in the activation and regulation of innate and adaptive immunity. Nature reviews Immunology. 2011;11(8):519–31. 10.1038/nri3024 . 21785456

[30] Mocsai A. Diverse novel functions of neutrophils in immunity, inflammation, and beyond. The Journal of experimental medicine. 2013;210(7):1283–99. 10.1084/jem.20122220 23825232 PMC3698517

[31] Kolaczkowska E, Kubes P. Neutrophil recruitment and function in health and inflammation. Nature reviews Immunology. 2013;13(3):159–75. 10.1038/nri3399 . 23435331

[32] Nauseef WM, Borregaard N. Neutrophils at work. Nature immunology. 2014;15(7):602–11. 10.1038/ni.2921 . 24940954

[33] Gabriel C, Her Z, Ng LF. Neutrophils: neglected players in viral diseases. DNA and cell biology. 2013;32(12):665–75. 10.1089/dna.2013.2211 . 24236425

[34] Stacey MA, Marsden M, Pham NT, Clare S, Dolton G, Stack G, et al Neutrophils recruited by IL-22 in peripheral tissues function as TRAIL-dependent antiviral effectors against MCMV. Cell host & microbe. 2014;15(4):471–83. 10.1016/j.chom.2014.03.003 24721575 PMC3989063

[35] Jenne CN, Wong CH, Zemp FJ, McDonald B, Rahman MM, Forsyth PA, et al Neutrophils recruited to sites of infection protect from virus challenge by releasing neutrophil extracellular traps. Cell host & microbe. 2013;13(2):169–80. 10.1016/j.chom.2013.01.005 . 23414757

[36] van Gisbergen KP, Sanchez-Hernandez M, Geijtenbeek TB, van Kooyk Y. Neutrophils mediate immune modulation of dendritic cells through glycosylation-dependent interactions between Mac-1 and DC-SIGN. The Journal of experimental medicine. 2005;201(8):1281–92. 10.1084/jem.20041276 15837813 PMC2213143

[37] Eken C, Gasser O, Zenhaeusern G, Oehri I, Hess C, Schifferli JA. Polymorphonuclear neutrophil-derived ectosomes interfere with the maturation of monocyte-derived dendritic cells. Journal of immunology. 2008;180(2):817–24. . 18178820 10.4049/jimmunol.180.2.817

[38] Puga I, Cols M, Barra CM, He B, Cassis L, Gentile M, et al B cell-helper neutrophils stimulate the diversification and production of immunoglobulin in the marginal zone of the spleen. Nature immunology. 2012;13(2):170–80. 10.1038/ni.2194 22197976 PMC3262910

[39] Charmoy M, Brunner-Agten S, Aebischer D, Auderset F, Launois P, Milon G, et al Neutrophil-derived CCL3 is essential for the rapid recruitment of dendritic cells to the site of Leishmania major inoculation in resistant mice. PLoS pathogens. 2010;6(2):e1000755 10.1371/journal.ppat.1000755 20140197 PMC2816696

[40] Bennouna S, Bliss SK, Curiel TJ, Denkers EY. Cross-talk in the innate immune system: neutrophils instruct recruitment and activation of dendritic cells during microbial infection. Journal of immunology. 2003;171(11):6052–8. . 14634118 10.4049/jimmunol.171.11.6052

[41] Morel C, Badell E, Abadie V, Robledo M, Setterblad N, Gluckman JC, et al Mycobacterium bovis BCG-infected neutrophils and dendritic cells cooperate to induce specific T cell responses in humans and mice. European journal of immunology. 2008;38(2):437–47. 10.1002/eji.200737905 . 18203135

[42] Duffy D, Perrin H, Abadie V, Benhabiles N, Boissonnas A, Liard C, et al Neutrophils transport antigen from the dermis to the bone marrow, initiating a source of memory CD8+ T cells. Immunity. 2012;37(5):917–29. 10.1016/j.immuni.2012.07.015 . 23142782

[43] Meri S, Pangburn MK. Discrimination between activators and nonactivators of the alternative pathway of complement: regulation via a sialic acid/polyanion binding site on factor H. Proceedings of the National Academy of Sciences of the United States of America. 1990;87(10):3982–6. 1692629 10.1073/pnas.87.10.3982 PMC54028

[44] Favoreel HW, Van de Walle GR, Nauwynck HJ, Pensaert MB. Virus complement evasion strategies. The Journal of general virology. 2003;84(Pt 1):1–15. . 12533696 10.1099/vir.0.18709-0

[45] Nakagawa K, Kitazume S, Oka R, Maruyama K, Saido TC, Sato Y, et al Sialylation enhances the secretion of neurotoxic amyloid-beta peptides. Journal of neurochemistry. 2006;96(4):924–33. 10.1111/j.1471-4159.2005.03595.x . 16412100

[46] Richards AA, Colgrave ML, Zhang J, Webster J, Simpson F, Preston E, et al Sialic acid modification of adiponectin is not required for multimerization or secretion but determines half-life in circulation. Molecular endocrinology. 2010;24(1):229–39. 10.1210/me.2009-0133 . 19855092 PMC5428139

[47] Lucas A, McFadden G. Secreted immunomodulatory viral proteins as novel biotherapeutics. Journal of immunology. 2004;173(8):4765–74. . 15470015 10.4049/jimmunol.173.8.4765

[48] Lalani AS, Masters J, Graham K, Liu L, Lucas A, McFadden G. Role of the myxoma virus soluble CC-chemokine inhibitor glycoprotein, M-T1, during myxoma virus pathogenesis. Virology. 1999;256(2):233–45. 10.1006/viro.1999.9617 . 10191189

[49] Lalani AS, Ness TL, Singh R, Harrison JK, Seet BT, Kelvin DJ, et al Functional comparisons among members of the poxvirus T1/35kDa family of soluble CC-chemokine inhibitor glycoproteins. Virology. 1998;250(1):173–84. 10.1006/viro.1998.9340 . 9770431

[50] Lalani AS, Graham K, Mossman K, Rajarathnam K, Clark-Lewis I, Kelvin D, et al The purified myxoma virus gamma interferon receptor homolog M-T7 interacts with the heparin-binding domains of chemokines. Journal of virology. 1997;71(6):4356–63. 9151824 10.1128/jvi.71.6.4356-4363.1997 PMC191652

[51] Mossman K, Nation P, Macen J, Garbutt M, Lucas A, McFadden G. Myxoma virus M-T7, a secreted homolog of the interferon-gamma receptor, is a critical virulence factor for the development of myxomatosis in European rabbits. Virology. 1996;215(1):17–30. 10.1006/viro.1996.0003 . 8553583

[52] Mossman K, Upton C, McFadden G. The myxoma virus-soluble interferon-gamma receptor homolog, M-T7, inhibits interferon-gamma in a species-specific manner. The Journal of biological chemistry. 1995;270(7):3031–8. . 7852384 10.1074/jbc.270.7.3031

[53] Upton C, Mossman K, McFadden G. Encoding of a homolog of the IFN-gamma receptor by myxoma virus. Science. 1992;258(5086):1369–72. . 1455233 10.1126/science.1455233

[54] Macen JL, Upton C, Nation N, McFadden G. SERP1, a serine proteinase inhibitor encoded by myxoma virus, is a secreted glycoprotein that interferes with inflammation. Virology. 1993;195(2):348–63. 10.1006/viro.1993.1385 . 8337817

[55] Audry M, Jeanneau C, Imberty A, Harduin-Lepers A, Delannoy P, Breton C. Current trends in the structure-activity relationships of sialyltransferases. Glycobiology. 2011;21(6):716–26. 10.1093/glycob/cwq189 . 21098518

[56] Rakic B, Rao FV, Freimann K, Wakarchuk W, Strynadka NC, Withers SG. Structure-based mutagenic analysis of mechanism and substrate specificity in mammalian glycosyltransferases: porcine ST3Gal-I. Glycobiology. 2013;23(5):536–45. 10.1093/glycob/cwt001 . 23300007

