# Supplementary material for: The α2,3-Sialyltransferase Encoded by Myxoma Virus Is a Virulence Factor that Contributes to Immunosuppression
Source: PLoS One. 2015 Feb 23;10(2):e0118806. doi: 10.1371/journal.pone.0118806 (PMC4338283; doi:10.1371/journal.pone.0118806)
Supplement: S1 Fig — NZW rabbits were inoculated with 100 PFUs of MYXV M138L Del or M138L STOP strains in the left flank or left uninfected (Mock). 100 days p.i., rabbits of all groups were challenged with the inoculation of 100 PFUs of the MYXV WT strain. A. Daily clinical scores that evaluated the physical condition and clinical signs of primary and systemic infection were obtained. Clinical scores in the Mock vaccinated group were recorded until animals reached euthanasia criteria (significant respiratory distress, or no food or water intake for 48 h, or a clinical score of 15 for two consecutive days). B. The results were analyzed for survival rates among the groups. The daily percentage of survival in each group was plotted to generate the survival curve. The data were analyzed by log-rank (Mantel-Cox) test, * p<0.05. C. Size of the primary lesions of NZW rabbits from the different groups. Daily measurements of the lesion area were recorded. The data presented are the average +/- SEMs. (PDF) [file pone.0118806.s001.pdf]

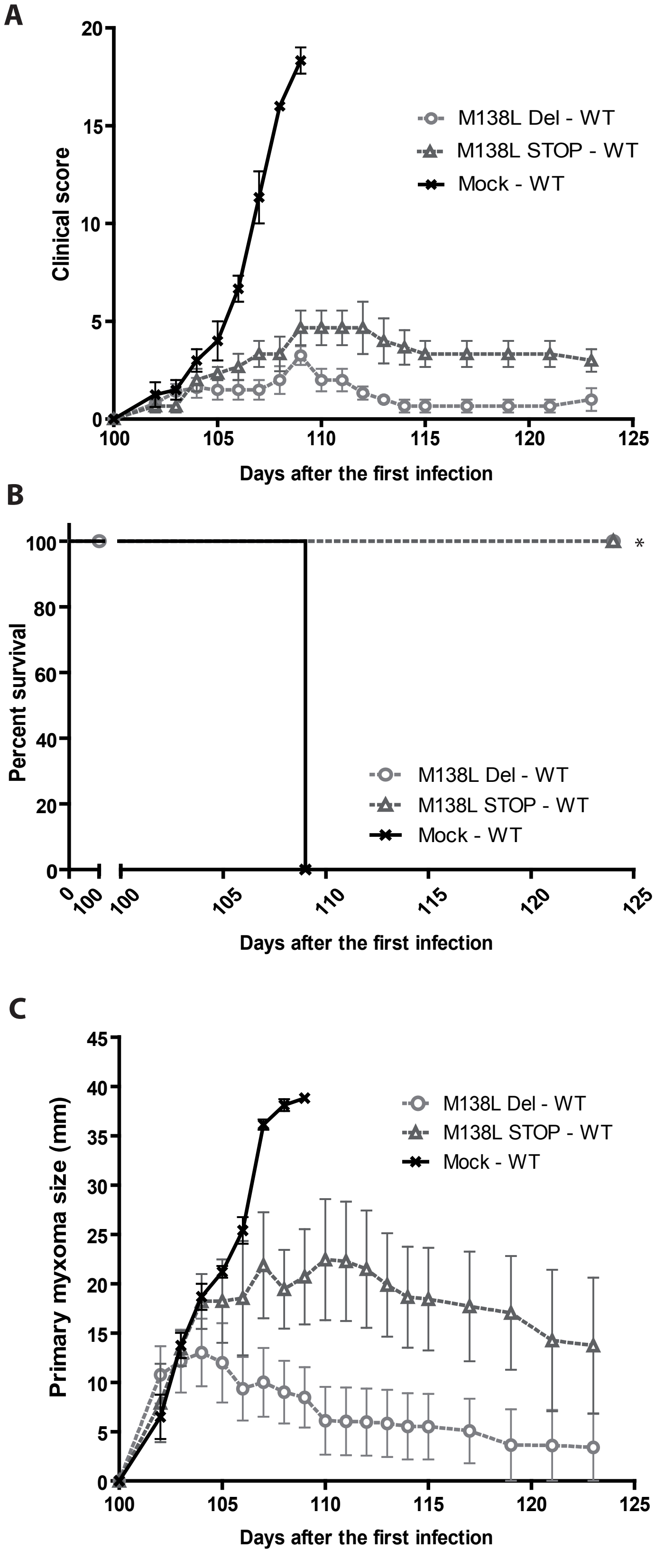

Figure S1: Protection of the rabbits infected with the Myxoma virus (MYXV) M138L Del and STOP strains against a challenge with the WT strain. NZW rabbits were inoculated with 100 PFUs of MYXV M138L Del or M138L STOP strains in the left side or left uninfected (Mock). 100 days p.i., rabbits of all groups were challenged with the inoculation of 100 PFUs of the MYXV WT strain. **A.** Daily clinical scores that evaluated the physical condition and clinical signs of primary and systemic infection were obtained. Clinical scores in the Mock vaccinated group were recorded until animals reached euthanasia criteria (significant respiratory distress, or no food or water intake for 48 h, or a clinical score of 15 for two consecutive days). **B.** The results were analyzed for survival rates among the groups. The daily percentage of survival in each group was plotted to generate the survival curve. The data were analyzed by log-rank (Mantel-Cox) test, * $p<0.05$. **C.** Size of the primary lesions of NZW rabbits from the different groups. Daily measurements of the lesion area were recorded. The data presented are the average +/- SEMs.
